# Practical Guidance for Interventions in Adults with Metabolic Syndrome: Diet and Exercise vs. Changes in Body Composition

**DOI:** 10.3390/ijerph16183481

**Published:** 2019-09-18

**Authors:** Enrique Albert Pérez, Marina Poveda González, Rosa María Martínez-Espinosa, Mariola D Molina Vila, Manuel Reig García-Galbis

**Affiliations:** 1 Faculty of Health Sciences, University of Alicante, 03690 Alicante, Spain; ejalbertperez@gmail.com (E.A.P.); marina.poveda94@gmail.com (M.P.G.); 2 Division of Biochemistry and Molecular Biology, Department of Agrochemistry and Biochemistry, Faculty of Sciences. University of Alicante, 03690 Alicante, Spain; rosa.martinez@ua.es; 3 Department of Mathematics, Faculty of Sciences, University of Alicante, 03690 Alicante, Spain; mariola.molina@ua.es; 4 Department of Nutrition and Dietetics, Faculty of Health Sciences, University of Atacama, Avda. Copayapu 2862, III Region, Copiapó 1530000, Chile

**Keywords:** metabolic syndrome, diabetes, diet, exercise, body composition, weight and fat

## Abstract

(1) Objective: to establish practical guidance for the design of future clinical trials in MS (metabolic syndrome) patients aged 18 and older, based on a systematic review of randomized clinical trials connecting diet, physical exercise and changes in body composition. (2) Method: this systematic review of randomized clinical trials (RCT) is based on the guidelines recommended by PRISMA (Preferred Reporting Items for Systematic reviews and Meta-Analyses). Criteria of selection: ≥18 years of age; patients diagnosed with MS; intervention programs including diet, physical exercise and/or modifications in the style of life as treatment, as well as the magnitude of changes in body composition (BC); randomized clinical trial published between 2004 and 2018. (3) Results: the multidisciplinary interventions describe major changes in BC, and the recurring pattern in these clinical trials is an energy reduction and control in the percentage of intake of macronutrients along with the performance of regularly structured exercise; the most analyzed parameter was waist circumference (88.9% of the trials), followed by body weight (85.2%), BMI (77.8%) and body fat (55.6%). (4) Conclusions: The analysis of the information here reported sheds light for the design of future clinical trials in adults with MS. The best anthropometric parameters and units of measurement to monitor the interventions are related to dietary and physical exercise interventions. A list of practical advice that is easy to implement in daily practice in consultation is here proposed in order to guarantee the best results in changes of body composition.

## 1. Introduction

### 1.1. Metabolic Syndrome (MS): Concept, Prevalence and Diagnostic Criteria

MS was firstly described in 1920 by Kylin, a Swedish physician, as a connection between hypertension, hyperglycemia and gout. Later, in 1947, Vague indicated that visceral obesity is commonly associated with metabolic alterations, mentioning cardiovascular diseases and type 2 diabetes mellitus (T2DM) as the key pathologies. Then in 1965, at the annual meeting of the European Association for the Study of Diabetes, MS was described as a syndrome related to hypertension, hyperglycemia and obesity. In 1989, Kaplan linked the syndrome to the combination of upper-body obesity, glucose intolerance, hypertriglyceridemia and hypertension. However, in 1998, a WHO diabetes research group defined MS as a group of interconnected physiological, biochemical, clinical and metabolic factors that directly increase the risk of cardiovascular disease, T2DM and all-cause mortality [1]. The MS is also known as “insulin resistance syndrome”, “quartet of death” or “syndrome X” [2], thus including abdominal obesity, prehypertension or hypertension, dyslipidemia and prediabetes [3]. Useful definitions for the readers to distinguish between diabetes, prediabetes, insulin resistance, etc. can be found in the review of the American Diabetes Association [4].

This syndrome affects to more than 20% of the adult population of the United States, China, Europe and the developed countries. This prevalence increases with age in a specific correlation with sex: before the age of 50 years, men have a higher prevalence and after 50 years, this trend is reversed [5,6,7]. There are up to five types of diagnostic criteria to identify MS [1,8], consequently the lack of unification in the universally accepted diagnostic criteria makes it difficult to determine the prevalence of MS [9,10]. Thus, the diagnostic criteria of the MS change depending on the age (few criteria exist for MS diagnosis of children, teenagers and adults) and the continent [1,8,11].

### 1.2. MS: Causes, Diet, Exercise and Anthropometric Parameters

The main factors promoting MS and consequently T2DM are: excess ingestion of nutrients, low physical activity and the production of inflammatory cytokines [12,13,14,15,16]. As an example, in an investigation including 61,239 men and 73,216 women (40–74 years of age) from Shanghai (China), it was observed that with major fulfilment of the dietetic directives, minor mortality was detected [17]. The increase of physical exercise is the best non-pharmacological treatment for obesity, as it can reduce insulin resistance, to counteract the inflammatory state and to improve the lipid profile [7,18]. Changes of the following anthropometrical parameters could contribute to the improvement of health in MS patients: fat mass index (fat mass·height^−2^), waist circumference, abdominal diameter and corporal weight [19,20,21,22].

This systematic review is justified by the following facts:

1. The high rates of MS worldwide and the lack of consensus in the criteria used for MS diagnosis make difficult to determine MS prevalence [1,8].
2. Dietary and physical exercise interventions are both causative and controlling tools for eliminating MS risk factors and the emergence of T2DM [11,12,13,14,15]. Thus, it is important to analyze the characteristics of the interventions causing the greatest changes [19,20,21,22].

The main objective of this systematic review was to establish practical guidance for the design of future clinical trials in MS patients aged 18 and older, based on a systematic review of randomized clinical trials connecting diet, physical exercise and changes in body composition. Other secondary objectives related to MS have also been analyzed: (a) to record which interventions (exclusive or multidisciplinary interventions) produce the greatest changes in body composition in MS patients ≥18 years of age; (b) to identify which are the dietetic and physical exercise patterns showing the most significant changes in corporal composition in MS patients ≥18 years of age, in order to stablish accurate clinical trials in the next future; (c) to identify the most used anthropometric parameters and units of measure to record the changes in body composition, in MS patients ≥18 years old; (d) to analyze dietary and physical exercise patterns proposed by guidelines for intervention in overweight, obesity, diabetes and MS patients ≥18 years of age.

## 2. Materials and Methods

### 2.1. Search Strategy and Information Processing

This systematic review of randomized clinical trials is based on the guidelines recommended by PRISMA (Preferred Reporting Items for Systematic reviews and Meta-Analyses) [23]. The information retrieval system “Boolean” was used to identify the works of interest for this review [24].

All the data used in this study were obtained from the following databases: CINAHL (Current Nursing and Allied Health Literature), ProQuest (which supplies services of information for universities, schools, public companies, corporations and public libraries worldwide), PubMed (a search engine for free access to the MEDLINE database of citations and abstracts of biomedical research articles) and Web of Science (online scientific information service, provided by Thomson Reuters, integrated in ISI Web of Knowledge, WOK, containing original articles based on clinical trials). The keywords used were: “MS” “weight loss”, “fat loss”, “diet”, “exercise” and “lifestyle”. These keywords were obtained from the “MeSH database” (Medical Subject Heading) and NLM (The National Library of Medicine). Controlled vocabulary thesaurus was used for indexing articles for PubMed. The search strategies used are displayed in Table 1. To identify and to select the information obtained from databases, the following filters were used: date of publication between January 2004 and December 2018. Other additional settings from each specific database were fixed: “CINAHL” database, “AB Summary” and “academic publications”; “ProQuest” database, advanced search including the options “evaluated by experts”, “scientific magazines”, “article” and “abstract”; “PubMed” database, advanced search including the options “Title/Abstract” and “clinical trial”; “Web of Science” database, basic search selecting the options “theme”, “article” and “clinical trial”. The starting open research questions were as follows. What interventions produce the greatest changes in body composition in MS patients ≥ 18 years of age? What dietetic and physical exercise patterns show the most significant changes in corporal composition? What are the most used anthropometric parameters and units of measure to record the changes in body composition? Is it possible to propose more accurate practical guidance in order to promote more significant changes in body composition?

### 2.2. Selection of the Articles Previously Identify

**Figure 1:**
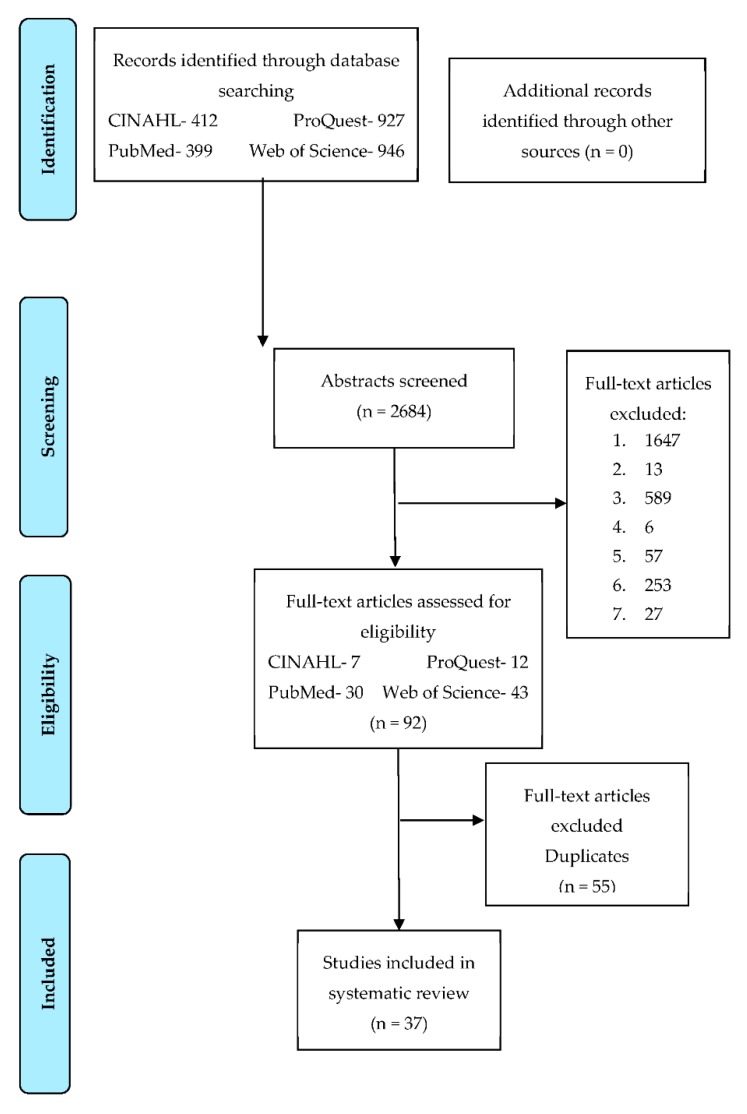
Flow chart in the screening process for the selection of included clinical trials [23]. Legend exclusion criteria: 1. they include one, or part of the main subjects considered for this review (MS, T2DM or insulin resistance); 2. diet and/or physical exercise are not included for weight loss; 3. They are not clinical trials, with different interventions and their comparison; 4. not being a randomized clinical trial; 5. the decrease of body weight, BMI, body fat or waist circumference are not analyzed at least before and after the intervention; 6. they include in the sample patients under 18 years of age; 7. studies carried out with animal models instead of human beings.

Each of the identified articles were independently analyzed by three researchers. PICOS strategy was used to define the eligibility criteria for this review (population, intervention, comparisons, results and characteristics of clinical trials):

Different criteria included in the search strategy were: age of the patients ≥18 years [25]; patients with MS [1,8]; intervention programs including diet, physical exercise and/or modifications in the style of life as treatment as well as the magnitude of changes in body composition; randomized clinical trial published between 2004 and 2018 in scientific journals in Spanish and English (Figure 1). The two languages were selected due to their impact and use at global scale (English is in general the “lingua franca” for communicating science. Besides, English and Spanish are two of three most spoken languages worldwide (https://danivoiceovers.com/en/los-10-idiomas-mas-hablados-mundo/; https://www.europapress.es/sociedad/noticia-idiomas-cifras-cuantas-lenguas-hay-mundo-20190221115202.html). The articles finally selected summarized details about the changes in body composition through the anthropometric parameters and units of measure (Table 2 and Table 3) [26,27,28,29,30,31,32,33,34,35,36,37,38,39,40,41,42,43,44,45,46,47,48,49,50,51,52,53,54,55,56,57,58,59,60,61,62].

A total amount of 2684 articles were discarded due to the following exclusion criteria: they include one, or part of the main subjects considered for this review (MS, T2DM or insulin resistance) (1647 articles, 61.4%); diet and/or physical exercise are not included for weight loss (13 articles, 0.48%); they are not a clinical trial, with different interventions and their comparison (589 articles, 21.94%); they are not randomized clinical trials (six articles, 0.22%); the decrease of body weight, BMI, body fat or waist circumference are not analyzed at least before and after the intervention (57 articles, 2.21%); they include in the sample patients under 18 years of age (253 articles, 9.43%); studies carried out with animal models instead of human beings (27 articles; 1.01%); clinical trials repeated (55 articles; 2.05%) (Figure 1) [23]. Besides, it was also necessary to discard two intervention groups from two independent clinical trials, because other methods of changes in body composition such as the gastric ball and supplementation were included as a subsection of intervention (Table 3) [48,59].

Data were extracted from the following five domains [23] (Table 2 and Table 3):

1. Population: characteristics of the population studied (country of origin, type of diagnostic criteria, number, age and gender), inclusion and exclusion criteria.
2. Interventions: exclusive and multidisciplinary as therapeutic treatments.
3. Comparators: inclusion of randomized clinical trials, control and intervention groups are identified. In principle, only the intervention groups receive the therapeutic treatment that should cause changes in body composition.
4. Results: identified as variation in body composition, presenting significant and not significant variations.
5. Characteristics of clinical trials: authors, year of publication, type of randomized clinical trial, duration of intervention, instrument of analysis of body composition, type of intervention used (exclusive or multidisciplinary) and body composition variation (measured with different anthropometric parameters and units of measurement).

### 2.3. Data Analysis, Identification of Information Loss Risks

All the essential information required to carry out this systematic review is summarized in a total of six tables, a flowchart and a figure. The flowchart and Table 1 display the details of the search strategies and databases used; Table 2 and Table 3 display the relevant details of the interventions recorded including the following items: title, author(s), type of sample/group, duration of the intervention, type of method/intervention, variation of the anthropometric parameters and units of measurement (this variation was registered using the following parameters): body weight (BW) in kg; body fat (BF) in kg or %; body mass index (BMI) in kg/m^2^ and waist circumference (WC) in cm, or their respective drop in percentage (%) in each parameter. To identify the variation of these parameters and units, a Yes/No code has been used: YES means that the article includes the study of the parameters in the respective standard units, and NO means that the article does not include the parameters in their study. Thus, the most significant changes in body composition from each clinical trial was registered based on each parameter and unit (Table 2 and Table 3).

The searches were made independently (one for each of the authors). The other tables were performed jointly.

Heterogeneity of the clinical trials design has been the major limitation in this research. Only few articles present adequate data to calculate heterogeneity statistics, but the contrasts done in this sense with the analyzed works reported, in general, a lack of homogeneity.

In order to analyze the quality in the design of clinical trials included in this work involving the highest variations in body composition, the Consort method has been used (assessment and implementation guide on the most appropriate guidelines for the design of randomized trials). Negative results should be considered for items that have not been performed or have been performed in an incomplete way, and positive results are those in which the items were fulfilled entirely [63]. The results of this analysis have been summarized with the calculation of the percentage of negative results compared to the totality of analyzed items. The designs in this review indicate low positive results so this would be one of the limitations of this research.

## 3. Results

### 3.1. Search Features and Types of Interventions Identified

A total of 2684 articles were identified, but finally only 1.6% of the overall were included on the basis on the search strategy (Figure 1). The most useful strategy search included the following variables: “metabolic syndrome” and “weight loss” or “weight reduction” or “fat loss” or “fat reduction” or “lifestyle” and “exercise” or “physical activity” or “sport” or “weightlifting”; Web of Science and PubMed are the databases that had the most clinical trials included (Table 1). After analyzing the items, they were classified into two groups according to the type of intervention used: exclusive intervention (27%, Table 2) and multidisciplinary interventions (73%, Table 3). The most relevant characteristics of the clinical trials included are (Table 2 and Table 3): number of patients from 21 to 406 (median, 49 patients); greater proportion of mixed samples (75.7%), 8.1% only constituted by men and 16.2% only constituted by women; patients from 18 to 80 years old; sample groups located in Europe (35.1%), America (29.7%), Asia (18.9%) and Oceania (16.2%); Adult Treatment Panel III (ATPIII) of the National Cholesterol Education Program (NCEP), International Diabetes Federation (IDF), World Health Organization (WHO) and The Examination Committee of Criteria for “Metabolic Syndrome” in Japan as MS diagnosis criteria; temporary duration of the studies from 0.75 to 36 months (median and mode 3 months, up to 6 months, 81.1%). The most abundant intervention modalities observed were diet, physical exercise and/or changes in lifestyle (a combination of diet and physical exercise predominates as intervention (45.9% of the studies)).

Regarding the ten works based on exclusive interventions, the duration of the interventions in eight of them ranged from 2.5 to 6 months and just one had a duration up to 24 months. The sample size ranged from 27 to 173 patients (median, 93.5 patients). Eight of them assigned the patients into two groups (two intervention groups in five works and an intervention group together with a control group in the others three). In the two remaining works, the patients were assigned into two intervention groups and a control group. Concerning to the method of intervention, there were three works with modifications in lifestyle, four impinged changes in diet and three controlled the method of exercise.

Regarding the twenty-seven articles based on multidisciplinary interventions, the duration in the interventions ranged from 0.75 to 36 months (77.8% with a period up to 6 months; five and one with durations of 12 and 36 months, respectively). The sample size ranged from 21 to 406 patients (median, 48 patients). Fourteen of them assigned the patients into two groups (two intervention groups in nine works and an intervention group together with a control group in the others five). Eleven works designated three groups (nine studies with two intervention groups and a control group) and finally, there were two works with four intervention groups. Concerning the methods of intervention, there were seventeen works with interventions on diet and exercise and ten with modifications in diet, exercise and lifestyle.

### 3.2. The Most Significant Body Composition Changes in MS (Table 2 and Table 3)

As a response to the first secondary objective, the analysis reveals that multidisciplinary interventions report the greatest changes in body composition. In this context, Luley and co-workers show the largest changes in BW, BMI and WC [58]. This was a 12-month multidisciplinary intervention in diet, exercise and lifestyle, carried out in Europe with a sample of 178 patients (mainly men, 57%) between 30 and 60 years of age, randomized in a control group and two intervention groups. All the subjects were advised to increase their usual daily physical activity following Magdeburg Dual Diet (500 kcal per day and low in carbohydrates, preferably with a low glycemic index). Diet and exercise were monitored in IG1 weekly by letter, in IG2 monthly by telephone, whereas CG was not monitored. The changes of each variable measured was: 12.2 kg (CI 95%, 10.5–13.8), (11.4% (9.8–12.9)), 4.1 kg/m^2^ (3.6–4.6) (12%) and 14.3 cm (12.3–16.2) (12.1%) in IG1 for BW, BMI and WC, respectively.

The Malin and Bonfanti clinical trials, based on multidisciplinary intervention techniques, show the best results for reducing BF [39,43]. On the other hand, the clinical trial of Bonfanti and coworkers is a 3-month European study involving patients of both sexes, over 50 years old (n = 36) and randomized in four intervention groups [39]. In two groups, the subjects followed hypocaloric diets with different proportion in carbohydrates, protein and fats and in the remaining groups exercise was also added. Subjects in IG1 followed a Mediterranean diet while subjects in IG2 followed a low fat diet rich in complex carbohydrates. In IG3 and IG4, the respective diets of IG1 and IG2 were combined with aerobic exercise. The change of BF was 12% in IG4.

It was analyzed whether the included clinical trials that have obtained the greatest variations in body composition have developed the items recommended by the CONSORT method [63]. Between the studies here included the following three have reported the highest number of positives results: Luley et al., Malin et al. and Bonfanti et al. (59.4%, 37.84% and 35.2%, respectively) [39,43,58].

As a response to the second secondary objective, energy restriction in diet was a common characteristic of the clinical trials that achieved the greatest changes in body composition in this review, occasionally combined with structured physical exercise.

### 3.3. Anthropometric Parameters and Units of MS Changes in Body Composition Measurements (Table 2 and Table 3)

**Figure 2:**
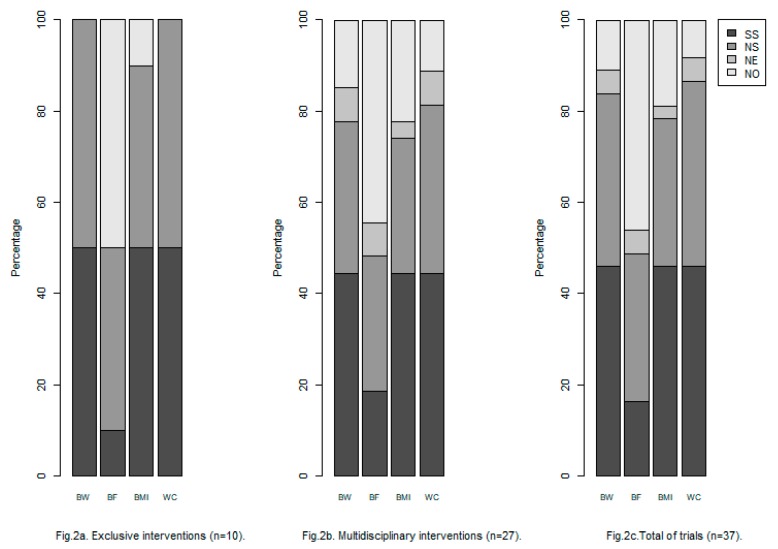
Percentage of articles that studied the parameter analyzed in this systematic review and results of comparison between groups. body weight (BW); body fat (BF); body mass index (BMI); control group (CG); intervention group (IG); non-significant (NS); statistically significant (SS); the information is not available in the clinical trial evaluated (NE); the article does not include its study (NO); waist circumference (WC).

With regard to the articles based on exclusive interventions, all of them study and report a statistically significant (*p*-value <0.05) changes in BW within the intervention groups (final vs. baseline levels), with the exception of an intervention group in which no significant differences in BF, BMI and WC were observed [32]. Regarding to the differences in BW between intervention groups, they turned out to be statistically significant in 50% of the total studies (Figure 2a).

In the case of BF, 50% of the works had results expressed in kg or percentage. Only one of them presented statistically significant differences between the analyzed groups. On the other hand, nine of the ten trials studied the changes in BMI in kg/m^2^, obtaining statistically significant changes among groups in five of the nine studies. Finally, the loss of cm in WC was collected in all the works, reporting statistically significant changes in four of the ten trials.

Concerning multidisciplinary intervention trials (Figure 2b), 85.2% of them included the study of BW changes in kg or/and relative units. All but three of the trials studied and reported a statistically significant (*p*-value <0.05) changes in BW (kg or relative units) within the intervention groups (final vs. baseline levels), except in [36,37,39]. Related to the differences in BW between intervention groups, they turned out to be statistically significant (*p*-value <0.05) in 52.2% of the cases (Figure 2b).

The variable BF was not included in 44.4% of the works analyzed, neither in absolute terms (kg or %) nor in relative units. Fourteen studies reported significant differences within the intervention groups but eight studied reported non-significant differences between the intervention groups.

Concerning BMI, twenty-one works included the study of the changes in absolute units and one of them incorporates their study in relative units. Among these articles, twelve had significant differences between groups. Finally, the loss in WC (in cm) was collected in 88.9% of the works, registering statistically significant changes in twelve of the twenty-four trials. The study of the relative changes appeared in three studies, with statistically significant differences between intervention groups in one of them.

In order to reach the last secondary objective of this review, from all the articles selected it is possible to conclude that the most analyzed parameter was WC (88.9% of the trials), followed by BW (85.2%), BMI (77.8%) and BF (55.6%). Regarding WC, 44.4% of the articles reported statistically significant differences among groups, 37% reported non-significant differences and 7.4% did not specify the classification of the differences. In the case of BW, BMI and BF the percentages were 44.4%, 33.3% and 7.4%, 44.4%, 29.6% and 3.7% and 18.5%, 29.6% and 7.4%, respectively (Figure 2c).

## 4. Discussion

Due to the high rates of MS worldwide [5,6,7] and the fact that the guidelines for MS interventions are scarce and old (dietary and physical exercise interventions) [64,65,66], it is necessary to delve into which practical guidance would report a greater variation of body composition. In order to reach the fourth secondary objective, Table 4 and Table 5 were done [67,68,69,70,71,72,73,74] (European Guidelines and institutional guides such as: AACE, ADA, ACE, AHA, NHLBI). The selected guides had to meet the following criteria: to have a degree of evidence analysis; the main objective was to cause changes in body composition; review articles would have been published in journals indexed in the Journal Citation Reports (Web of Science) between 2005 and 2018.

The reasoning of using the most appropriate practical guidance for obesity and T2DM in order to address the lack of knowledge in MS is: the appearance of abdominal obesity is the most frequent in this syndrome [13,75,76,77], and the known relationship of developing this syndrome at the same time as diabetes [14,15,78]. With all this in mind and as result of this work, the following advices for the design of efficient interventions in adults with MS are proposed:

To obtain the greatest changes produced in body composition, it is recommended multidisciplinary interventions (Table 2 and Table 3) and Table 4 indicates 5 factors that should be intervened: medical nutrition therapy, regular physical activity, sleeping between 6 and 9 h sleep, limiting alcohol intake to moderate, and cessation of smoking (at the end of the procedure).

Several options have been identified in the energy constraint to be applied to dietary intake: 40% energy reduction progressively [39] or a restriction of 500 kcal day^−1^ [58] (Table 3) vs. restriction of 1000 kcal day^−1^ and energy intake (1200–1500 kcal day^−1^ in women and 1500–1800 kcal day^−1^ in men) (Table 5). Considering all the reports’ approaches, we suggest a restriction between 500 and 1000 kcal day^−1^. The nutritionist should be cautious in the degree of energy restriction that is prescribed and adapt to the individualization of each subject, because there are different tolerances to fasting situations. Individuals with insulin-resistant excess weight compared with thin subjects have metabolic inflexibility (they did not register this adaptation to use fuel for the oxidation of fatty acids). This inflexibility is associated with several pathologies (metabolic syndrome, diabetes mellitus type 2 and cancer) and several factors such as: composition of the diet, frequency of ingestion, physical exercise and the use of certain pharmacological compounds [79,80].

Two options are presented in the distribution of macronutrients: (i) 55–58% carbohydrates, less than 30% fat and 15–22% proteins [39,43]; (ii) low in carbohydrates with a low glycemic index [58] (Table 3) vs. advices summarized in Table 5.

Aerobic exercise between 65–85% intensity daily (maximum heart rate) is recommended [39,44] (Table 3) vs. guidelines (Table 5) which recommend 150 min wk^−1^ (minutes per week) (3 to 5 days wk^−1^) and introduces strength training. The prescription for physical exercise must be structured (Table 5) [22]. The concepts of structured physical exercise were identified by the American College of Sports Medicine [81].

The reduction of 5% of body weight is advised, which will lead to improvements in metabolic alterations [30,82]. Despite the above, it would be more advisable to indicate the variation of 5% of body fat instead, in subjects with obesity and/or MS. This limit would be valid for the following cases: (i) subjects performing physical exercise, for the increase of muscle mass affecting body weight; (ii) for the reduction of subjects with low excess weight or fat (overweight and/or high fat level); (iii) and to set an accessible target for subjects with higher excess weight and fat (obesity and very high fat level) [83].

On the other hand, the following complementary advices are also established:

To follow the guidelines for the design of randomized trial reports [63] and other randomized guides [84].

The nutritionist involved should be the manager of the design of the dietetic intervention [71,85,86].

To incorporate body composition measuring devices for clinical and research use on MS [86,87]. The following anthropometric parameters should be used for the calculation of metabolic risk: fat mass index (fat mass height^−2^), waist circumference, abdominal diameter and body weight [19,20,21,22]. The main predictor of adverse metabolic events is visceral adipose tissue [75,76,88,89].

The tools used for evaluation of the nutritional condition at the beginning and in its follow-up must be described as follows.

- The available procedures must be identified to recognize in what type of patients and in what moment they must be applied. The variation in the use of the procedures can be due to factors as: ethnic groups, age, gender and physical limitations (in case of the use of crutches, prothesis and/or wheelchair or even in patients without toes due to amputations because of the diabetes) [71,86].
- The use of distance or remote health care is recommendable: (i) when the patient does not reside in the same population of the center of health and/or hospital [86]; (ii) in case of older people or specific cases in which the patients find hard to attend themselves to the center [86]; (iii) in coaching strategy [90,91].
- Monitoring of physical exercise and/or daily physical activity is recommended (triaxial accelerometers) [92].
- It is recommended to extrapolate the concept “obesogenic” for MS patients. This concept was firstly used in 1996 (“obese” means excess of corporal fat and “genic” refers to production or synthesis) [93]. Currently, the obesogenic environmental is defined as “the addition of the influences that have the environments, the opportunities or the living conditions to promote the obesity in individuals or populations” [94]. These influences promote the consumption of high levels or energy as well as diets based on non-healthy food (fast food, take away meals, etc.) [95].
- Coaching is highly recommended as part of the design of further clinical trials with MS patients. In the coaching per pairs approach during interventions of overweight, obesity and diabetes patients, several instructed patients are selected to accompany and monitor to other patients suffering the same pathology. Some recent studies have revealed that thanks to the coaching per pairs approach, the levels of glycosylated hemoglobin, cholesterol, LDL, blood pressure, corporal mass index (CMI), relation waist—hip, selfcare activities in the case of diabetes, depression and other quality life factors are positively modified [90,91,96,97].
- The nutritionist should be focused on “how” the patient or the family can apply the recommendations given by professionals. The intervention must be considered not only for the professional but also for the patient as a dynamic process, in which it will be necessary to overcome unforeseen challenges [86,98].

As a new contribution, the authors of this work extrapolate the theory of the training cycle program of an athlete to the terminology in this process of learning how to apply to lifestyle modification, improving theoretical and practical applications [99,100]. The learning system of the improvement of the alimentary habits must turn into a learning in the modification of the style of life (diet and exercise). In this system, three periods or blocks are differentiated (initiation, improvement and maintenance) (Table 6).

The professional should locate a subject in one period or another as follows. In the initiation period, three cases could be possible: (a) The treatment has just begun; (b) the patient is reincorporated to this period because they have increased body fat in several consecutive consultations; (c) the patient rejoins because the treatment was previously abandoned. Advancing or remaining in the improvement period occurs in two cases: a) they are in the initiation stage and body fat is decreasing regularly until there is a considerable decrease (≥5%), depending on each case; (b) the patient rejoins after the maintenance period because for personal reasons the treatment was paused at this stage. They will advance or remain in the maintenance period in the event that they have assimilated the recommendations to modify your lifestyle and is aware that they should not return to the previous habits in order not to relapse into the variation of body fat, having or not having reached healthy body fat level (≥10%, cumulative of the initiation and improvement stage), depending on each case [86].

## 5. Conclusions

The discrepancies found in the recommendations reported in previous works indicate that more research is required in this area of knowledge. From this systematic review, it can be concluded that the best anthropometric parameters and units of measurement to monitor the interventions are those related to dietary and physical exercise interventions. Besides, new practical guidance and advices are introduced in order to improve further clinical interventions. Thus, this systematic review will help with daily clinical practice in public health, sport sciences, nutrition and dietetics as well as endocrinology and metabolism, because the practical guidance here proposed guarantee the best results in changes of body composition, being easy to implement in daily practice in the consultation. It will therefore help the professional and patient to identify easily and quickly how the treatment should develop and what characteristics it should have. In addition, they are practical advices established based on studies in which the best results are obtained in terms of changes in body composition. The implementation of practical advices will help the public health on a large scale if the decrease of the body composition also diminishes the metabolic alterations (thus lowering the cost of healthcare).

## Figures and Tables

**Table 1:** Search strategies used to identify and select clinical trials, dates: 2004–2018.

Search Strategy	CINAHL Identified/Included	ProQuest Identified/Included	PubMed Identified/Included	Web of Science Identified/Included
“metabolic syndrome” AND “weight loss” OR “weight reduction” OR “fat loss” OR “fat reduction” OR “lifestyle” AND “exercise” OR “physical activity” OR “sport” OR “weightlifting”	222/5	532/7	187/22	303/29
“metabolic syndrome” AND “weight loss” OR “weight reduction” OR “fat loss” OR “fat reduction” OR “lifestyle” AND “diet” OR “dietary treatment” AND “feeding” OR “nutrition” OR “nutritional counselling”	27/1	87/2	18/2	149/6
“type II diabetes” OR “insulin resistance” AND “weight ls” OR “weight reduction” OR “fat loss” OR “fat reduction” AND “diet” OR “dietary treatment” OR “feeding” AND “nutrition” OR “nutritional counselling” OR “lifestyle”	44/0	77/0	42/2	155/3
“type II diabetes” OR “insulin resistance” AND “weight loss” OR “weight reduction” OR “fat loss” OR “fat reduction” AND “exercise” OR “physical activity” OR “sport” OR “weightlifting”	119/1	231/3	152/4	339/5

Articles or clinical trials identified: complete list of articles retrieved from various databases (PubMed, Web of Science, etc.), without having made the selection of the clinical trials of interest. Articles or clinical trials included: articles that meet the selection criteria.

**Table 2:** Characteristics of the randomized trials included in the reduction of body composition in metabolic syndrome: exclusive interventions.

Author (s) [26,27,28,29,30,31,32,33,34,35]	Location	Study Design	MS Diagnosis Criteria [1]	Sample/Groups/Characteristics Studied	Duration (Months)	Body Composition Measurement Instrument	Intervention Method Characteristics Studied/Comparative Statistical Analysis of BC	Anthropometric Parameters and Measurement Unit Analyzed (Statistical Results)				Decreases in Body Composition Mean ± SD or Mean ± (SE) or Mean (CI, 95%)
								BW (kg/%)	BF (kg or %)	BMI (kg/m^2^/%)	WC (cm/%)	
[26]	Asia (Iran)	RCT	ATPIII	n = 87 IG1: 43 IG2: 44 Women: 60.95% Age: 45.5	2.5	Body composition indices were measured via bioelectric impedance analysis (BIA; In Body s10; Korea). Height was measured bar using a stadiometer	**Diet** - Within groups: IG1: low fat plain yogurt IG2: fortified yogurt - Between groups	**YES**/NO *p* < 0.001 *p* < 0.001 NS	**YES**/**YES** *p* < 0.0001 *p* < 0.05 *p* < 0.05	**YES**/NO *p* < 0.001 *p* < 0.001 NS	**YES**/NO *p* < 0.001 *p* < 0.001 *p* < 0.05	**BW:** IG1: 4.3 ± 1.9 kg IG2: 5.1 ± 3.0 kg **BF:** IG1: 1.7 ± 2.4 kg and 1.7 ± 3.2% IG2: 3.4 ± 4.7 kg and 3.0 ± 2.8% **BMI:** IG1: 1.6± 0.1 kg IG2: 1.8 ± 1.16 kg **WC:** IG1: 4.4 ± 1.7 cm IG2: 5.8 ± 2.0 cm
[27]	Asia (Israel)	RCT (parallel-arm)	ATPIII	n = 74 IG1: 38 IG2: 36 Women: 100% Age: 30–57	3	BW and WC were measured by using a scale model Detector Physician Beam Scale (HOSPEQ, Inc., Miami, FL) vs. the same person according to the guidelines of the National Heart, Lung, and Blood Institute respectively	**Diet**- Within groups: IG1 (high breakfast kcal content) IG2 (high dinner kcal content) - Between groups	**YES**/NO *p* < 0.0001 *p* < 0.05 *p* < 0.0001	NO/NO	**YES**/NO *p* < 0.0001 *p* < 0.05 *p* < 0.0001	**YES**/NO *p* < 0.0001 *p* < 0.05 *p* < 0.0001	**BW:** IG1: 8.7 ± (1.4) kg IG2: 3.6 ± (1.5) kg **BMI:** IG1: 3.1± (0.4) kg/m^2^ IG2: 1.3± (0.4) kg/m2 **WC:** IG1: 8.7 ± (0.9) cm IG2: 3.6 ± (0.9) cm
[28]	Europe (Spain)	RCT (parallel-group)	ATP III	n = 50 IG1: 25 IG2: 25 Men (56%) Age: 18–65	3	Height and BC was measured using a wall-mounted stadiometer vs. Tanita TBF-300 (Tanita Corp., Tokyo, Japan) bioimpedance analysis device respectively	**Diet** - Within groups: IG1 (nut diet) IG2 - Between groups	**YES**/NO *p* < 0.05 *p* < 0.05 NS	**YES** (%)/NO *p* < 0.05 *p* < 0.05 NS	NO/NO	**YES**/NO *p* < 0.05 *p* < 0.05 NS	**BW:** IG1: 2.2 kg (0.9–3.4) IG2: 1.5 kg (0.6–2.4) **BF:** IG1: 1.9% (0.9–2.5) IG2: 1.1% (0.3–1.9) **WC:** IG1: 3.8 cm (1.9–5.8) IG2: 2.7 cm (1.0–4.4)
[29]	Europe (Italy)	RCT	ATP III	n = 100 IG1: 50 IG2: 50 Women: 73% Age: >18	5	Height was measured using a stadiometer and WC, to the measurement of the narrowest circumference between the bottom of the rib cage and the iliac crest by using an unstretched tape measure	**Diet** - Within groups: IG1 (high CH diet) IG2 (low CH diet) - Between groups	**YES**/**YES** *p* < 0.001 *p* < 0.001 NS	NO/NO	**YES**/NO *p* < 0.001 *p* < 0.001 NS	**YES**/**YES** *p* < 0.001 *p* < 0.001 NS	**BW:** IG1: NE (kg) IG1: 10% IG2: NE (kg) IG2: 10% **BMI:** IG1: NE IG2: NE **WC:** IG1: NE (cm) IG1: 8% IG2: NE (cm) IG2: %
[30]	Europe (Spain)	RCT (Randomized-block)	IDF	n = 160 IG: 138 CG: 22 Women: 50% Age: 54 ± 8	4	Body weight was assessed in an electronic scale (Hawk, Mettler Toledo, USA) body composition was determined by dual energy X-ray absorptiometry (DXA Hologic Series Discovery Wi QDR, Bedford, USA). WC was measured in a horizontal plane 2 cm above the iliac crest	**Exercise** Within groups: IG: aerobic interval training CG: sedentary Between groups	**YES**/NO *p* < 0.05 NE *p* < 0.05	**YES**/NO NS NE NS	**YES**/NO *p* < 0.05 NE *p* < 0.05	**YES**/NO *p* < 0.05 NE *p* < 0.05	**BW:** IG: 1.2 kg CG: increase **BF:** IG: 0.7 kg CG: increase **BMI:** IG: 0.4 kg CG: increase **WC:** IG: 2.6 cm CG: increase
[31]	North America (USA)	RCT	IDF	n = 34 CG: 10 IG1:13 IG2: 11 Women: 75% Age: 49.1 ± 1.8	4	Air displacement plethysmography (Bod-Pod; Life Measurement Instruments, Concord, CA)	**Exercise** - Within groups: IG1 (low intensity exercise) IG2 (high intensity) - Between groups	**YES**/NO *p* < 0.01 *p* < 0.01 NS	**YES** (%)/NO *p* < 0.01 *p* < 0.01 NS	**YES**/NO *p* < 0.01 *p* < 0.01 NS	**YES**/NO NS *p* < 0.01 NS	**BW:** IG1: 2.7 kg IG2: 2.7 kg **BF:** IG1: 0.7% IG2: 1.6% **BMI:** IG1: 0.9 kg/m^2^ IG2: 0.9 kg/m^2^ **WC:** IG1: 2 cm IG2: 4.9 cm
[32]	North America (USA)	RCT	IDF	n = 27 CG: 7 IG1: 11 IG2: 9 Women: 100% Age: 51 ± 9	4	Air displacement plethysmography (Bod-Pod, Life Measurement Instruments, Concord, CA)	**Exercise** - Within groups: IG1 (low-intensity aerobic) IG2 (moderate-to-high intensity aerobic) - Between groups: IG1 vs. IG2 IG1 vs. CG IG2 vs. CG	**YES**/NO NS *p* < 0.05 NS NS NS	**YES** (%)/NO NS *p* < 0.05 NS NS NS	**YES**/NO NS *p* < 0.05 NS NS NS	**YES**/NO NS *p* < 0.05 *p* < 0.05 NS *p* < 0.05	**BW:** IG1: 2.1 kg IG2: 3.5 kg **BF:** IG1: 0.4% IG2: 1.7% **BMI:** IG1: 0.8 kg/m^2^ IG2: 1.3 kg/m^2^ **WC:** IG1: 1.2 cm IG2: 5.6 cm
[33]	Asia (China)	RCT	IDF	n = 173 IG: 86 CG: 87 Women: 50.8% Age: 24–78	3	NE	**Intervention in lifestyle** - Within groups: IG (Lifestyle intervention program) - Between groups	**YES**/NO NE *p* < 0.01	NO/NO	**YES**/NO NE *p* < 0.01	**YES**/NO NE NS	**BW:** IG: 1.77 kg CG: increase **BMI:** IG: 0.58 kg/m^2^ CG: 0.01 kg/m^2^ **WC:** IG: 3.69 (male)/1.37 (female) CG: 1.61 (male)/1.34 (female)
[34]	North America (USA)	RCT	IDF	n = 135 IG1: 72 IG2: 63 Women: 75% Age: 52	24	The International Diabetes Federation definition requires central obesity, measured by WC with ethnicity-based cutoffs	**Intervention in lifestyle** - Within groups: IG1 (individual counselling) IG2 (group counselling) - Between groups	**YES**/**YES** *p* < 0.05 *p* < 0.05 *p* < 0.001	NO/NO	**YES**/NO *p* < 0.001 *p* < 0.05 *p* < 0.05	**YES**/NO *p* < 0.05 *p* < 0.05 NS	**BW:** IG1: 2.2 ± 14.2 kg IG1: 1.8 ± 18.6% IG2: 6.2 ± 14.3 kg IG2: 5.6 ± 26.8% **BMI:** IG1: 0.8 kg/m^2^ IG2: 2.1 kg/m^2^ **WC:** IG1: 2.4 ± 15.5 cm IG2: 3.1 ± 15.5 cm
[35]	Asia (East Asia)	RCT	The Examination Committee of Criteria for “Metabolic Syndrome” in Japan	n = 102 IG:49 CG:53 Men: 100% Age: 53.2 ± 6.8	6	Body height and body weight were measured using an automated scale (AD-6225A; A&D, Tokyo, Japan)	**Intervention in lifestyle** CG (standard healthy recommendations) - Within groups: IG (intervention in lifestyle) - Between groups	**YES**/NO *p* < 0.001 *p* < 0.001	NO/NO	**YES**/NO *p* < 0.001 *p* < 0.01	**YES**/NO *p* < 0.001 *p* < 0.05	**BW:** IG: 2.01 kg **BMI:** IG: 0.6 kg/m^2^ **WC:** IG: 2.51 cm

Body weight (BW); body fat (BF); body mass index (BMI); waist circumference (WC); control group (CG); intervention group (IG); carbohydrates (CH); non-significant (NS); the information is not available in the clinical trial evaluated (NE); randomized controlled trial (RCT); YES: the article includes the analysis of the parameter expressed in its correspondent units; NO: the article does not include the analysis of the parameter.

**Table 3:** Characteristics of the randomized trials included in the reduction of body composition in metabolic syndrome: multidisciplinary interventions.

Author(s) [36,37,38,39,40,41,42,43,44,45,46,47,48,49,50,51,52,53,54,55,56,57,58,59,60,61,62]	Location	Study Design	MS Diagnosis Criteria [1]	Sample/Groups/Characteristics Studied	Duration (months)	Body Composition Measurement Instrument	Intervention Method/Comparative Statistical Analysis of the BC	Anthropometric Parameters and Measurement Unit Analyzed (Statistical results)				Decreases in Body Composition Mean ± SD or Mean ± (SE) or Mean (CI, 95%)
								BW (kg/%)	BF (kg or %)	BMI (kg/m^2^/%)	WC (cm/%)	
[36]	North America (USA)	RCT	NE	n = 39 IG1:23 IG2:16 Women: 60% Age: 38–76	6	NE	**Diet, exercise** - Within groups: IG1 (hypocaloric diet + MUFA) IG2 (hypocaloric diet + PUFA) - Between groups	**YES**/NO NS *p* < 0.01 NE	NO/NO	NO/NO	NO/NO	**BW:** IG1: 2.3 kg ± (1) IG2: 4.6 kg ± (2)
[37]	Europe (Spain)	RCT	NE	n = 36 CG: 12 IG1:12 IG2: 12 Men: 75% Age: 54 ± 9	4	Dual Energy X-ray absorptiometry scans (Hologic Discovery DXA Series Wi QDR, Bedford, USA)	**Diet, exercise** - Within groups: IG1 (EXER-then-DIET) IG2 (EXER+DIET) - Between groups: IG1 vs. CG IG2 vs. CG IG2 vs. IG1	**YES**/**YES** NS *p* < 0.05 NE NE NE	**YES**/NO NS *p* < 0.05 NE NE NE	**YES**/NO NS *p* < 0.05 NE NE NE	**YES**/NO *p* < 0.05 *p* < 0.05 NS *p* < 0.05 NS	**BW:** IG1: NE kg IG1: NE% IG2: NE kg IG2: 5.5 ± 0.8% **BF:** IG1: NE (kg) IG2: NE (kg) **BMI:** IG1: increase IG2: 1.8 kg/m^2^ **WC:** IG1: NE (cm) IG2: NE (cm)
[38]	Oceania (Australia)	RCT	ATPIII	n = 62 IG1: 21 IG2: 20 IG3: 21 Women: 55% Age: 30–60	6	The participants weighing < 157 kg was measured with Hologic DXA (QDR-4500W; Hologic Corporation), and those weighing > 157 kg were measured with a GE Lunar iDXA (General Electric)	**Diet, exercise** - Within groups: IG1 (M-DASH diet) IG2 (BOLD diet) IG3 (BOLD + diet) - Between groups	**YES**/NO *p* < 0.001 *p* < 0.001 *p* < 0.001 NS	**YES**/NO *p* < 0.001 *p* < 0.001 *p* < 0.001 NS	**YES**/NO *p* < 0.001 *p* < 0.001 *p* < 0.001 NS	**YES**/NO *p* < 0.001 *p* < 0.001 *p* < 0.001 NS	**BW:** IG1: 5.1 kg IG2: 4.8 kg IG3: 4.8 kg **BF:** NE **BMI:** IG1: 1.8 kg/m^2^ IG2: 0.5 kg/m^2^ IG2: 1.7 kg/m^2^ **WC:** IG1: 5.5 cm IG2: 7.6 cm IG3: 6.2 cm
[39]	Europe (Spain)	RCT	ATP III	n = 36 IG1: 8 IG2: 8 IG3: 10 IG4: 10 Women and Men: NE Age: 50–70	3	The anthropometric study stated by the International Society for the Advancement of Kit anthropometry (method ISAK); to evaluate BW and height rods were used (MB 201T Bonus); the cutaneous folds were valued by means of the Harpenden calibrator	**Diet, exercise** Within groups: IG1 (Hypocaloric Med diet) IG2 (Low fat-high CH diet) IG3 (Hypocaloric Med diet and exercise) IG4 (Low fat-high diet and exercise) - Between groups: IG2 vs. IG1 IG2 vs. IG3 IG2 vs. IG4 IG4 vs. IG1 IG4 vs. IG3	NO/**YES** NS *p* < 0.05 NS *p* < 0.05 *p* < 0.05 *p* < 0.05 *p* < 0.05 *p* < 0.05 *p* < 0.05	NO/**YES** *p* < 0.05 *p* < 0.05 *p* < 0.05 *p* < 0.05 *p* < 0.05 *p* < 0.05 *p* < 0.05 *p* < 0.05 *p* < 0.05	NO/NO	NO/NO	**BW:** IG1: ~ 7% IG2: ~ 8% IG3: ~ 5% IG4: ~ 10% **BF:** IG1: ~ 7% IG2: ~ 10% IG3: ~ 9% IG4: ~ 12%
[40]	Europe (Austria)	RCT	ATPIII	n = 71 IG1: 36 IG2: 35 Men: 100% Age: 36–66	0.75	NE	**Diet, exercise** - Within groups: IG1 (moderate altitude exercise) IG2 (sea level exercise) - Between groups	**YES**/NO *p* < 0.001 *p* < 0.001 NS	**YES**/NO *p* < 0.001 *p* < 0.001 NE	**YES**/NO *p* < 0.001 *p* < 0.001 NS	**YES**/NO *p* < 0.001 *p* < 0.001 NE	**BW:** IG1: 3.22 ± 1.91 kg IG2: 3.04 ± 2.16 kg **BF:** NE **BMI:** IG1: 0.81 kg/m^2^ IG2: 0.69 kg/m^2^ **WC:** NE
[41]	Europe (France)	RCT	OMS	n = 78 IG1: 24 IG2: 24 IG3: 30 Women: 56% Age: 50–70	12	DXA (Hologic QDR 4500 series; Waltham, USA)	**Diet, exercise** - Within groups: IG1 (moderate-resistance and moderate-endurance) IG2 (high-resistance and moderate-endurance) IG3 (moderate-resistance and high-endurance) - Between groups	NO/**YES** *p* < 0.001 *p* < 0.001 *p* < 0.001 NS	NO/**YES** *p* < 0.001 *p* < 0.001 *p* < 0.001 NS	NO/NO	NO/**YES** *p* < 0.001 *p* < 0.001 *p* < 0.001 NS	**BW:** All: 6.3 ± 7.2% IG1: 5.9 ± 5.8% IG2: 8.4 ± 8.9% IG3: 4.7 ± 6.7% **BF:** All: 1.7 ± 1.7% IG1: 1.8 ± 1.5% IG2: 2.1 ± 2.3% IG3: 1.3 ± 1.3% **WC:** All: 7.7 ± 6.2% IG1: 7.7 ± 6.6% IG2: 9.5 ± 6.8% IG3: 6.3 ± 5%
[42]	Europe (Spain)	RCT	ATPIII	n = 40 IG1: 20 IG2: 20 Women: 67% Age: 50–66	3	NE	**Diet, exercise** - Within groups: IG1 IG2 (exercise) - Between groups	**YES**/**YES** *p* < 0.05 *p* < 0.05 *p* < 0.05	**YES** (%)/NO *p* < 0.05 *p* < 0.05 *p* < 0.05	**YES**/NO *p* < 0.05 *p* < 0.05 *p* < 0.05	**YES**/NO *p* < 0.05 *p* < 0.05 NS	**BW:** IG1: 5.38 kg IG1: 6.23 ± (0.83)% IG2: 8.38 kg IG2: 8.45 ± (0.76)% **BF:** IG1: 2.76% IG2: 4.5% **BMI:** IG1: 2.09 kg/m^2^ IG2: 3.26 kg/m^2^ **WC:** IG1: 3.72 cm IG2: 4.18 cm
[43]	North America (USA)	RCT	ATP III	n = 21 IG1: 11 IG2: 10 Men: 52% Age: 66.2 ± 1.1	3	Dual-x-ray absorptiometry (DEXA; Lunar Prodigy, Madison, WI); Height was measured with a wall-mounted stadiometer and weight was recorded on a digital scale in a hospital gown	**Diet, exercise** - Within groups: IG1 (high glycemic diet) IG2 (low glycemic diet) - Between groups	**YES**/NO *p* < 0.001 *p* < 0.001 NS	**YES**/NO *p* < 0.001 *p* < 0.001 NS	**YES**/NO *p* < 0.001 *p* < 0.001 NS	NO/**YES** *p* < 0.05 *p* < 0.05 *p* < 0.05	**BW:** IG1: 11 kg IG2: 6.8 kg **BF:** IG1: 8.9 kg IG2: 5.7 kg **BMI:** IG1: 3.6 kg/m^2^ IG2: 2.7 kg/m^2^ **WC:** IG1: ~10% IG2: ~6%
[44]	Oceania (Australia)	RCT	IDF	n = 58 IG1: 20 IG2: 19 CG: 19 Men: 59% Age: 55 ± 6	3	Dual-energy X-ray absorptiometry (DXA, GE-LUNAR Prodigy Advance PA+130510, GE Medical Systems, Lunar, Madison, WI, USA)	**Diet, exercise** - Within groups: IG1 (diet) IG2 (diet and exercise) - Between groups: IG1 vs. IG2 IG1 vs. CG IG2 vs. CG	**YES**/NO *p* < 0.001 *p* < 0.001 NS *p* < 0.01 *p* < 0.01	**YES**/NO *p* < 0.001 *p* < 0.001 NS *p* < 0.01 *p* < 0.01	**YES**/NO *p* < 0.001 *p* < 0.001 NS *p* < 0.01 *p* < 0.01	**YES**/NO *p* < 0.001 *p* < 0.001 NS *p* < 0.05 *p* < 0.01 *p* < 0.01	**BW:** IG1: 7.1 ± 2.9 kg IG2: 8.7 ± 4.6 kg **BF:** IG1: 5.2± 3.0 kg IG2: 7.0± 3.9 kg **BMI:** IG1: 2.4± 1.0 kg/m^2^ IG2: 2.9± 1.4 kg/m^2^ **WC:** IG1: 6.7±3.2 cm IG2: 10.0±5.2cm
[45]	North America (USA)	RCT (parallel-arm)	ATPIII	n = 32 IG1: 8 IG2: 9 IG3: 8 IG4: 7 Men: 100% Age: 59 ± 7	3	Tanita BC-418 Segmental Body Composition Analyzer/Scale (Tanita Inc. Tokyo, Japan), which has been shown to correlate strongly (r ≥ 0.95, *p* < 0.001) with both whole-body and regional composition values obtained using the gold standard, dual-energy X-ray absorptiometry (DEXA)	**Diet, exercise** - Within groups: IG1 (low fat diet) IG2 (low fat diet and exercise) IG3 (carbohydrate-restricted diet) IG4 (carbohydrate-restricted diet and exercise) -Between groups	NO/NO	NO/NO	NO/NO	**YES**/NO *p* < 0.01 *p* < 0.01 *p* < 0.01 *p* < 0.01 NS	**WC:** IG1: 5 cm IG2: 10 cm IG3: 8 cm IG4: 10 cm
[46]	South America (Brazil)	RCT	ATPIII	n = 75 IG1: 25 IG2: 25 CG: 25 Men: 65% Age: 30–55	12	Bioelectrical impedance (Omron HBF 306 Bioimpedance Analyzer) and WC was measured between the last rib and the iliac crest	**Diet, exercise** CG (high CH diet and exercise recommendations) - Within groups: IG1 (low CH diet and walking) IG2 (low CH diet and aerobic exercise) -Between groups	**YES**/NO *p* < 0.001 *p* < 0.001 NS	**YES**/NO *p* < 0.001 *p* < 0.001 NS	**YES**/NO *p* < 0.001 *p* < 0.001 NS	**YES**/NO *p* < 0.001 *p* < 0.001 NS	**BW:** IG1: 9 kg IG2: 11 kg CG: 8 kg **BF:** IG1: 3% IG2: 3% CG: 2% **BMI:** IG1: 2.9 kg/m^2^ IG2: 3.5 kg/m^2^ CG: 2.9 kg/m^2^ **WC:** IG1: 14 cm IG2: 14 cm CG: 14 cm
[47]	Oceania (Australia)	RCT (parallel group)	ATPIII	n = 38 IG1: 13 IG2: 13 CG: 12 Women: 100% Age: 55 ± 1	3	DEXA scan (GE-LUNAR Prodigy Advance PAþ130510; GE Medical Systems, Lunar, Madison, Wisconsin, USA); BW, using a digital scale. WC at the midpoint between the lowest rib and iliac crest, and hip circumference at the level of the greater trochanters	**Diet, exercise** - Within groups: IG1 (diet) IG2 (diet and exercise) - Between groups: IG1 vs. IG2 IG1 vs. CG IG2 vs. CG	**YES**/**YES** *p* < 0.001 *p* < 0.001 *p* < 0.05 *p* < 0.01 *p* < 0.01	**YES**/NO *p* < 0.001 *p* < 0.001 *p* < 0.005 *p* < 0.001 *p* < 0.001	**YES**/NO *p* < 0.001 *p* < 0.001 NS *p* < 0.01 *p* < 0.00	**YES**/NO *p* < 0.001 *p* < 0.001 *p* < 0.01 *p* < 0.001 *p* < 0.001	**BW:** IG1: 7.9 ± (0.8) kg IG1: 8.2 ± (0.8)% IG2: 10.4 ± (1.1) kgIG2: 10.7 ± (0.8)% **BF:** IG1: 5.7 ± (0.9) kg IG2: 8.5 ± (1.0) kg **BMI:** IG1: 2.7 ± (0.3) kg/m^2^ IG2: 3.4 ± (0.3) kg/m^2^ **WC:** IG1: 7.0 ± (0.8) cm IG2: 10.9 ± (1.2) cm
[48]	North America (USA)	RCT	ATPIII	n = 24 IG1: 12 IG2: 12 Women: 83% Age: 25–80	3	NE	**Diet, exercise** - Within groups: IG1 (without interest, for the use of supplementation) IG2 (Mediterranean diet, exercise) - Between groups	NO/**YES** *p* < 0.01 *p* < 0.01 NS	NO/NO	**YES**/**YES** *p* < 0.01 *p* < 0.01 NS	NO/**YES** *p* < 0.01 *p* < 0.01 NS	**BW:** IG1: 6.8 ± (1.1)% IG2: 5.2 ± (1.1)%**BMI:** IG1:2.1 ± (0.3) kg/m^2^ IG1: 6.7± (0.9)% IG2: 1.8 ± (1.1) kg/m^2^ IG2: 5.2 ± (1.1)% **WC:** IG1: 5.9 ± (1.1)% IG2: 5.4 ± (1.1)%
[49]	Oceania (Australia)	RCT	ATPIII	n = 59 IG1: 20 IG2: 20 CG: 19 Men: 59% Age: 55 ± 1	3	Dual-energy X-ray absorptiometry scan (GE-LUNAR Prodigy Advance PA+130510; GE Medical Systems, Lunar, Madison, WI); BW was measured using a digital scale and WC was measured at the midpoint between the lowest rib and iliac crest and hip circumference at the level of the greater trochanters	**Diet, exercise** - Within groups: IG1 (diet) IG2 (diet and exercise) - Between groups: IG1 vs. IG2 IG1 vs. CG IG2 vs. CG	**YES**/**YES** *p* < 0.001/NE *p* < 0.001/NE NS/NS *p* < 0.01/NE *p* < 0.01/NE	**YES**/NO *p* < 0.001 *p* < 0.001 NS	**YES**/NO *p* < 0.001 *p* < 0.001 NS *p* < 0.01 *p* < 0.01	**YES**/NO *p* < 0.001 *p* < 0.001 *p* < 0.01 *p* < 0.01 *p* < 0.01	**BW:** IG1: 7.1 ± (0.6) kg IG1: 7.6 ± (0.7)% IG2: 8.4 ± (0.1) kg IG2: 8.7 ± (0.9)% **BF:** IG1:5.2 ± (0.7) kg IG2: 6.9 ± (0.9) kg **BMI:** IG1:2.4 ± (0.2) kg/m^2^ IG2: 2.8 ± (0.3) kg/m^2^ **WC:** IG1: 6.7 ± (0.7) cm IG2: 9.8 ± (1.2) cm
[50]	Oceania (Australia)	RCT	ATPIII	n = 34 IG1: 15 IG2: 19 Men: 62% Age: 55 ± 1	3	NE	**Diet, exercise** - Within groups: IG1 (exercise) IG2 - Between groups	**YES**/NO *p* < 0.001 *p* < 0.001 NS	**YES**/NO *p* < 0.001 *p* < 0.001 NS	NO/NO	**YES**/NO *p* < 0.001 *p* < 0.001 NS	**BW:** All: 8.2 ± (0.7) kg IG1: 8.4 ± (1.1) kg IG2: 8.1 ± (0.9) kg **BF:** All: 6.4 ± (0.6) kg IG1: 6.9 ± (1.1) kg IG2: 6.0 ± (0.7) kg **WC:** All: 8.6 ± (0.8) cm IG1: 9.8 ± (1.3) cm IG2: 7.6 ± (0.9) cm
[51]	North America (USA)	RCT	ATPIII	n = 24 IG1: 12 IG2: 12 Women: 62.5% Age: 65.5 ± 5.0	3	Hydrostatic weighing, and fat mass and fat-free mass were estimated using the equation of Siri	**Diet, exercise** - Within groups: IG1 IG2 (diet) - Between groups	**YES**/NO *p* < 0.001 *p* < 0.001 *p* < 0.05	**YES**/NO *p* < 0.001 *p* < 0.001 NS	**YES**/NO *p* < 0.001 *p* < 0.001 *p* < 0.05	**YES**/NO *p* < 0.001 *p* < 0.001 NS	**BW:** IG1: 3.7 ± 3.4 kg IG2: 6.8 ± 2.7 kg **BF:** IG1: 6.0 kg IG2: 4.5 kg **BMI:** IG1: 1.3 kg/m^2^ IG2: 2.4 kg/m^2^ **WC:** IG1: 5.6 cm IG2: 6.5 cm
[52]	North America (USA)	RCT (parallel-arm)	ATPIII	n = 47 IG1: 24 IG2: 23 Men: 50% Age: 20–65	3	DXA (QDR-4500W; Hologic Corp, Waltham, MA); BW by electronic scale (model CN20; Cardinal/Detecto, Webb City, MO); WC was measured according to guidelines of the National Heart, Lung, and Blood Institute (NHLBI)	**Diet, exercise** - Within groups: IG1 (without refined grain diet) IG2 (refined grain diet) - Between groups	**YES**/NO *p* < 0.001 *p* < 0.001 NS	**YES** (%)/NO *p* < 0.001 *p* < 0.001 NS	NO/NO	**YES**/NO *p* < 0.001 *p* < 0.001 NS	**BW:** IG1: 3.7 ± 3.5 kg IG2: 5.3 ± 5.2 kg **BF:** IG1: 1.2 ± 1.3% IG2: 1.0 ± 1.6% **WC:** IG1: 2.5 ± 3.7 cm IG2: 4.7 ± 6.4 cm
[53]	Asia (Thailand)	RCT	IDF	n = 110 IG1: 52 IG2: 58 Women: 83% Age: 42.5 ± 1.1	3	Body composition were measured using BIA (TANITA^®^ BC-418, Tanita corp., Tokyo, Japan). WC was measured using a no stretchable tape with measurement taken at a horizontal line midway between the highest point of iliac crest and the lowest ribs	**Diet, intervention in lifestyle** Within IG1: Lifestyle intervention IG2: lifestyle intervention plus meal replacement Between groups:	**YES**/NO *p* < 0.01 *p* < 0.01 *p* < 0.05	**YES**/**YES** *p* < 0.01 *p* < 0.01 NS	**YES**/NO *p* < 0.01 *p* < 0.01 *p* < 0.05	**YES**/NO *p* < 0.01 *p* < 0.01 NS	**BW:** IG1: 1.4 kg IG1: 1.53% IG2: 2.3 kg IG2: 2.86% **BF:** IG1: 1.05 kg IG1: 0.8% IG2: 1.58 kg IG2: 1% BMIIG1: 0.49 kg/m^2^ IG2: 0.94 kg/m^2^ WC IG1: 2.5 cm IG2: 3.25 cm
[54]	Asia (Iran)	RCT	ATPIII	n = 117 IG: 64 CG: 53 Men: 66.3% Age: 44.2 (s.d. = 10.0)	6	BW by a calibrated scale (Seca, Hamburg, Germany model 8811021658) to the nearest of 0.1 kg; Height by stadiometer (Seca, Hamburg, Germany) to the nearest of 0.1 cm	**Diet, intervention in lifestyle** - Within groups: IG (My Healthy Heart Profile interactive web) - Between groups	**YES**/NO *p* < 0.001 *p* < 0.05	NO/NO	**YES**/NO *p* < 0.001 NS	NO/NO	**BW:** IG: 4 kg **BMI:** IG: 1.2 kg/m^2^
[55]	Europe (Greece)	RCT	ATP III	n = 47 CG: 13 IG1: 16 IG2: 18 Men: 57% Age: 49.0 ± 11.8	6	Weight and height were measured on a leveled platform scale and a wall-mounted stadiometer, to the nearest 0.5 kg and 0.5 cm; WC was measured in the middle between the 12^th^ rib and the iliac crest	**Diet, intervention in lifestyle** CG (usual care) - Within groups: IG1 (face-to-face) IG2 (telephone group) - Between groups: IG1 vs. CG IG2 vs. CG	NO/NO	NO/NO	**YES**/NO *p* < 0.001 *p* < 0.001 *p* < 0.001 *p* < 0.001	**YES**/NO *p* < 0.001 *p* < 0.001 *p* < 0.001 *p* < 0.05	**BMI:** CG: 0.1 ± 1.0 kg/m^2^ IG1: 1.4 ± 1.5 kg/m^2^ IG2: 1.2 ± 1.4 kg/m^2^ **WC:** CG: 0.5 ± 4.4 cm IG1: 4.1 ± 5.0 cm IG2: 3.5 ± 4.4 cm
[56]	South America (Brazil)	RCT	ATP III	n = 58 CG: 17 IG1: 21 IG2: 20 Women: 55.5% Age: 30–59	3	Body weight measured, using a properly calibrated 160 kg Cauduro scale; WC, with a millimeter no extensible long tape at the abdomen’s maximum extension	**Diet, exercise, intervention in lifestyle** CG: (standard intervention) - Within groups: IG1 (group intervention) IG2 (individual intervention) - Between groups: IG1 vs. CG IG2 vs. CG IG1 vs. IG2	NO/NO	NO/NO	**YES**/NO *p* < 0.01 *p* < 0.05 *p* < 0.05 *p* < 0.05 NS	**YES**/NO *p* < 0.05 *p* < 0.05 *p* < 0.05 *p* < 0.05 NS	**BMI:** IG1: 1.8 kg/m^2^ IG2: 1.5 kg/m^2^ **WC:** IG1: 4.4cm IG2: 5.3cm
[57]	Europe (Spain)	RCT	IDF	n = 406 IG: 230 CG: 176 Men: 55% Age: 18–80	36	NE	**Diet, exercise, intervention in lifestyle** CG: 176 (healthy diet with general physical activity) - Within groups: IG (Mediterranean diet and exercise) - Between groups	**YES**/NO NE NS	NO/NO	**YES**/NO NE NS	**YES**/NO NE *p* < 0.001	**BW:** IG: increase **BMI:** IG: increase **WC:** IG: 0.3 ± 6.0 cm
[58]	Europe (Germany)	RCT (parallel-group) groups	IDF	n = 178 CG: 60 IG1: 60 IG2: 58 Men: 57% Age: 30–60	12	NE	**Diet, exercise, intervention in lifestyle** Within groups: IG1 (monitored weekly)IG2 (monitored monthly) - Between groups: IG1 vs. IG2 IG1 vs. CG IG1 vs. CG	**YES**/NO *p* < 0.001 *p* < 0.001 *p* < 0.05 *p* < 0.001 *p* < 0.01	NO/NO	**YES**/NO *p* < 0.001 *p* < 0.001 *p* < 0.05 *p* < 0.001 *p* < 0.01	**YES**/NO *p* < 0.001 *p* < 0.001 *p* < 0.05 *p* < 0.001 *p* < 0.05	**BW:** IG1: 12.2 kg (8.8–10.5) IG1: 11.4% (9.8–12.9) IG2: 8.8 kg (7.1–10.4) IG2: 8.6% (7–10.2) **BMI:** IG1: 4.1 kg/m^2^ (3.6–4.6) IG2: 2.8 kg/m^2^ (2.3–3.4) **WC:** IG1: 14.3 cm (12.3–16.3) IG2: 10.8 cm (8.9–12.8)
[59]	Oceania (Australia)	RCT	ATP III	n = 66 IG: 31 CG: 35 Women: 68% Age: 18–60	12	Tanita BC-418 segmental body composition analyzer (Tanita Corporation of America Inc., Arlington Heights, IL). WC was measured midway between the top of the iliac crest and the most inferior part of the rib cage	**Diet, exercise, intervention in lifestyle** IG: without interest, for the use of the gastric balloon - Within groups: CG (diet and exercise)	**YES**/**YES** *p* < 0.05	NO/NO	**YES**/NO NE	**YES**/NO NE	**BW:** CG: 5.3 kg CG: 5.2% **BMI:** CG: 1.9 kg/m^2^ **WC:** CG: 6.4 cm
[60]	Europe (Greece)	RCT	ATP III	n = 88 CG: 29 IG1: 29 IG2: 30 Men: 57% Age: 49.9 ± 10.8	6	BW and height were measured on a levelled platform scale and a wall-mounted stadiometer	**Diet, exercise, intervention in lifestyle** - Within groups: IG1 (healthy food) IG2 (healthy food and decrease of less healthy food) - Between groups: IG1 vs. CG IG2 vs. CG	NO/NO	NO/NO	**YES**/NO *p* < 0.05 *p* < 0.05 *p* < 0.05 *p* < 0.05	**YES**/NO *p* < 0.05 *p* < 0.05 *p* < 0.05 *p* < 0.05	**BMI:** CG: 0.1 ± 1.0 kg/m^2^ IG1: 1.2 ± 1.4 kg/m^2^ IG2: 1.2 ± 1.4 kg/m^2^ **WC:** CG: 0.4 ± 4.4 cm IG1: 3.5 ± 4.4 cm IG2: 3.1 ± 4.0 cm
[61]	Asia (South Korea)	RCT	ATPIII	n = 48 IG: 27 CG: 21 Women: 100% Age: 62.7 ± 9.0	12	BW was measured with a high-precision scale (InBody 220; Biospace company, Seoul, Korea); WC was measured midway between the lowest rib and the iliac crest	**Diet, exercise, intervention in lifestyle** - Within groups: IG - Between groups	**YES**/NO *p* < 0.001 *p* < 0.001	NO/NO	**YES**/NO *p* < 0.001 *p* < 0.001	**YES**/NO *p* < 0.001 *p* < 0.001	**BW:** IG: 4.3 kg **BMI:** IG: 1.4 kg/m^2^ **WC:** IG: 9.4 cm
[62]	Asia (South Korea)	RCT	ATPIII	n = 29 IG: 16 CG: 13 Women: 100% Age: 66.7 ± 9.7	1	BW was measured with a high-precision scale (GM1000; Neo GMTEC, Seoul, Korea); WC was measured midway between the lowest rib and the iliac crest	**Diet, exercise, intervention in lifestyle** - Within groups: IG - Between groups	**YES**/NO *p* < 0.001 *p* < 0.001	NO/NO	**YES**/NO *p* < 0.001 *p* < 0.001	**YES**/NO *p* < 0.001 *p* < 0.001	**BW:** IG: 4.6 kg **BMI:** IG: 2 kg/m^2^ **WC:** IG: 6.2 cm

Body weight (BW); body fat (BF); body mass index (BMI); waist circumference (WC); control group (CG); intervention group (IG); non-significant (NS); the information is not available in the clinical trial evaluated (NE); randomized controlled trial (RCT); **YES**: the article includes the analysis of the parameter expressed in its correspondent units; NO: the article does not include the analysis of the parameter; carbohydrate (CH); mono or poly-unsaturated fatty acids (MUFA, PUFA); first aerobic interval training, later diet (EXER-then-DIET); exercise and diet simultaneously (EXER + DIET); 18% proteins, mostly of vegetable origin, 55% carbohydrate and 27% fat (M-DASH); 18.4% proteins, highest proportion of animal origin, 54% carbohydrate and 27% fat (BOLD); 27% proteins, highest proportion of animal origin, 45% carbohydrate and 27% fat (BOLD+); physical exercise performed at a moderate height of 1700 m (moderate altitude exercise); exercise at sea level to 200 m (sea level exercise).

**Table 4:** Guidelines and consensus on the treatment of overweight, obesity, type 2 diabetes mellitus and metabolic syndrome: adults ^†^.

		Author [67,68,69,70,71,72,73,74]	Recommendations in Dietary Intervention and Exercise
**Overweight and Obesity**	**AACE/ACE**	[67]	**Dietary intervention:** energy reduction^†^ and several types of diets stated with indications for different macronutrients (section algorithm: lifestyle therapy); reducing total energy (caloric) intake should be the main component of any weight-loss intervention (grade A; BEL 1); even though the macronutrient composition of meals has less impact on weight loss than adherence rates in most patients. In certain patient populations, modifying macronutrient compositions may be considered to optimize adherence, eating patterns, weight loss, metabolic profiles, risk factor reduction, and/or clinical outcomes (grade A; BEL 1). **Physical exercise:** recommendations^†^; resistance training should be prescribed to patients with overweight or obesity undergoing weight-loss therapy to promote fat loss while preserving fat-free mass; involvement of an exercise physiologist or certified fitness professional in the care plan should be considered to individualize the physical activity prescription and improve outcomes (grade A; BEL 1). **Behavior interventions:** lifestyle therapy in patients with overweight or obesity should include behavioral interventions that enhance adherence to prescriptions for a reduced-calorie meal plan and increased physical activity; behavioral lifestyle intervention and support should be intensified if patients do not achieve a 2.5% weight loss in the first month of treatment, as early weight reduction is a key predictor of long-term weight-loss success (grade A; BEL 1). Degrees of evidence: Origin from American Association of Clinical Endocrinologists 2010 [68].
	**European Guidelines**	[69]	**Dietary intervention:** energy reduction^†^ (evidence, grade A, B); VLCDs^†^ are unsuitable as a sole source of nutrition for children and adolescents, pregnant or lactating women and the elderly (level 2); the combination of exercise with caloric restriction helps in reducing body weight and body fat and preserving FFM, as compared to diet alone (level 1; grade B) **Physical exercise:** recommendations^†^ (level 2; grade B) **BW:** A decrease of 5–15% over a period of 6 months is realistic and of proven health benefit (evidence, level 1)^†^ Levels and degrees of evidence. Origin: [70].
**T2DM**	**ADA**	[71]	**Dietary intervention:** energy reduction^†^ **Physical exercise:** recommendations^†^ (grade B) **BW:** in overweight and obesity with T2DM, a sustained reduction of 5%, improves glycemic control and reduces the need for glucose-lowering drugs; in obese patients with T2DM, weight loss >5% produces benefits in the control of blood glucose, lipids and blood pressure
	**AACE/ACE**	[72]	**Lifestyle therapy:** the key components are medical nutrition therapy, regular physical activity, enough hours of sleep (6–9 h), behavioral support, smoking cessation and avoidance of all tobacco products (this component should be left for the end of the intervention) (see Integral algorithm for the management of type 2 diabetes, prediabetes and obesity). Coulston and his colleagues present another approach in the definition of “medical nutrition therapy” [73].
**MS**		[74]	**Dietary intervention and physical exercise:** recommendations ^†^ **BW:** 7–10% reduction of body weight in a period of 6 to 12 months and achieve an ideal BMI < 25 kg m^−2^
	**AHA/NHLBI**	[64]	**Dietary intervention:** reduction of intake of saturated fat, trans fat, cholesterol **Physical exercise:** recommendations^†^ **BW:** 7–10% during the 1 year of therapy, continue with the goal of achieving a desirable weight (BMI < 25 kg m^−2^)

^†^ Extensive information is given in Table 5. American Association of Clinical Endocrinologist (AACE); American College of Endocrinology (ACE); American Diabetes Association (ADA); American Heart Association (AHA); best evidence level (BEL); is recommended for all people with prediabetes or DM, including T1D, T2D, GDM, and other less common forms of DM. MNT must be individualized, generally via evaluation and teaching by a trained nutritionist or registered dietitian or a physician knowledgeable in nutrition (Medical Nutrition Therapy); National Heart, Lung, and Blood Institute (NHLBI); Scottish Intercollegiate Guidelines Network (SIGN); type 2 diabetes mellitus (T2DM);very low energy density diets (VLCD); kilograms (Kg); percentage (%); body weight (BW); minutes (min); month (mo); week (wk); pounds (lb).

**Table 5:** Intervention strategies in the decrease of body composition, in overweight, obesity, type 2 diabetes mellitus and metabolic syndrome.

**Dietary Intervention**		
Energy Density Restriction/Energy Density recommends	Overweight and obesity	±500–750 kcal day^−1^ [66]; ± 600 kcal day^−1^ [69]; 800–1200 kcal day^−1^ [69]
	T2DM	±500–750 kcal day^−1^ [2017 [71]]/1200–1500 kcal day^−1^ in women and 1500–1800 kcal day^−1^ in men [71]
	MS	Reduction of 500–1000 kcal day^−1^ [74]
VLCD	Overweight and obesity	<800 kcal day^−1^ [69]
	T2DM and MS	ND
Macronutrients and diets	Overweight and obesity	Different amounts of macronutrients (hydrates, proteins and fats) and giving rise to different types of diets [66]; hypocaloric balanced diets result in clinically significant weight loss independent of macronutrients [69]
	T2DM and MS	Recommended diets may differ in advising foods high in fat or in hydrates; the recommendation for consumption of recommended foods does not differ: whole grains, vegetables, fruits, legumes, low-fat dairy products, lean meats, nuts and seeds; it is recommended to adapt the diet to the health status and the preferences of the patient [64,71,74]
**Physical Exercise**		
Overweight and obesity		Aerobic exercise > 150 min wk^−1^ (3 to 5 days wk^−1^) [67]; moderate aerobic exercise: 150 min wk^−1^ (energetically walking) [69]
T2DM		Greater than or equal to 150 min of physical exercise a week at moderate intensity, 3 days a week at least; In the US Department of Health, does not differentiate between indications for T1DM and T2DM [71]; 150 min wk^−1^ moderate effort; strength training and increase according to each individual [72]
MS		≥30 to 60 min of exercise (moderate intensity on most days of the week, according to each individual) [74]; ≥30 min from 5 days wk^−1^ continuous or intermittent (and preferably ≥60 min and moderate intensity) [64]

Metabolic syndrome (MS); minutes (min); not stated (ND); type 1 diabetes mellitus (T1DM); type 2 diabetes mellitus (T2DM); week (wk).

**Table 6:** Theory of the extrapolation of training cycle programs to the modification of lifestyle changes in metabolic syndrome with goals of body composition.

Lifestyle Modification Program [72]	Duration	Body Composition vs. Duration [83,98]	Comments [86]	Initial Assessment and Monitoring Questionnaires
Multiannual	Several years (2–4 years)	Must depend on excess of weight (corresponding to body fat).	In this program, the subject may evolve from one stage to another (initiation, improvement or maintenance).	Activity or exercise measurement: Triaxial accelerometers are recommended [92] Food ingestion [101,102] Alcohol consumption [103]
Macrocycle	Several months	Considerable reduction in body fat per stage (≥ 5%).	Each macrocycle must be identified by one stage, therefore one year may have several macrocycles.	To apply questionnaires or measuring instruments as much as necessary
Mesocycle	Several weeks	Objectives of body fat variation should initially be measured by the kilos, but over time, the percentage should be used as the most recommended unit of measure	Weekly planning: a. variation in energy intake, macronutrients; b. in physical exercise (Table 5) [81]; c. sleep quality; d. alcohol consumption.	
Microcycle	Several days	It is not recommended to use body composition measuring instruments.	It is important to measure dietary intake, physical exercise characteristics (Table 5), sleep hours and alcohol consumption (extra caloric intake)	
Routine of one or more consultation sessions	Several hours and minutes	The evolution of body composition, body fat and other anthropometric parameters will be analyzed.	A break in training lasting more than 40 min qualifies as two separate workouts.	

Extrapolation [99,100].
